# Minilaparoscopic Versus Conventional Laparoscopic Hysterectomy: Insights from a Single-Center Retrospective Cohort Study with Legal Considerations

**DOI:** 10.3390/medicina61071216

**Published:** 2025-07-03

**Authors:** Valentina Billone, Giuseppe Gullo, Eleonora Conti, Silvia Ganduscio, Sofia Burgio, Giovanni Baglio, Gaspare Cucinella, Lina De Paola, Susanna Marinelli

**Affiliations:** 1 Department of Obstetrics and Gynaecology, AOOR Villa Sofia–Cervello, University of Palermo, 90127 Palermo, Italy; valentina.billone@gmail.com (V.B.); .; gandusciosilvia@gmail.com (S.G.); gasparecucinella1@gmail.com (G.C.); 2 IVF Unit, Department of Obstetrics and Gynaecology, AOOR Villa Sofia-Cervello, University of Palermo, 90127 Palermo, Italy; 3 Italian National Agency for Regional Healthcare Services, 00187 Rome, Italy; baglio.giovanni@yahoo.it; 4 Department of Anatomical, Histological, Forensic and Orthopedic Sciences, Sapienza University of Rome, 00161 Rome, Italy; lina.depaola@uniroma1.it; 5 School of Law, Polytechnic University of Marche, 60121 Ancona, Italy

**Keywords:** laparoscopic hysterectomy, mini-laparoscopic hysterectomy, postoperative complications, professional liability

## Abstract

*Background and Objectives*: We compared mini-laparoscopic and laparoscopic hysterectomy in terms of surgery duration, postoperative pain, conversion rate, blood loss, postoperative complications (Clavien-Dindo classification), and the length of hospital stay. *Materials and Methods*: Patients were recruited between 1 January 2017 and 1 January 2024, at the Department of Gynecology, “Villa Sofia-Cervello” Hospital. Indications for hysterectomy included uterine myoma, endometriosis, endometrial hyperplasia, adenomyosis, high-grade cervical dysplasia, early-stage endometrial cancer, and microinvasive cervical cancer. Patients were divided according to treatment into conventional laparoscopic hysterectomy (LH) with all 5 mm ports or the needlescopic approach (minilaparoscopic hysterectomy [MLH]), using 3 mm instruments. Postoperative pain was assessed using the visual analog scale (VAS) at multiple time points (2, 6, 12, and 24 h post-surgery). *Results*: A total of 308 patients were enrolled, with 153 women in the LH group and 155 in the MLH group. The surgery duration was on average 105.5 min in LH and 98.8 min in MLH (*p* < 0.0001). The intraoperative blood loss averaged 195.1 mL in LH and 100.3 mL in MLH (*p* < 0.001). The average length of hospital stay was 4.0 days for women undergoing LH compared to 3.2 days for women undergoing MLH (*p* < 0.001). *Conclusions*: This retrospective study demonstrated that MLH is an effective and functional technique for treating various gynecological conditions, with advantages in terms of aesthetic outcomes and reduced perioperative pain and recovery times. The positive results, supported by key parameters such as surgical duration, blood loss, and complications, could serve as a foundation for future studies on larger populations and for improving clinical practices in gynecology.

## 1. Introduction

Hysterectomy is the most common gynecologic surgical procedure performed in women of reproductive age. According to data from the American College of Obstetricians and Gynecologists (ACOG), the most frequent indications for hysterectomy include symptomatic uterine fibroids (51.4%), abnormal uterine bleeding (41.7%), endometriosis (30%), and pelvic organ prolapse (18.2%) [1]. By age 60, approximately one in three women in the United States will have undergone a hysterectomy [2]. Based on the underlying pathology, as well as the patient’s needs and desires, and in accordance with guidelines, the hysterectomy procedure can be of different types. It may be a total hysterectomy, in which the entire uterus and the fallopian tubes are removed; it can be a total hysterectomy with removal of the uterus, cervix, fallopian tubes, and ovaries; or it may be a subtotal hysterectomy, in which the body of the uterus is removed, leaving the cervix in place. Hysterectomy can be performed using different surgical techniques. Laparoscopic hysterectomy is the procedure for the removal of the uterus performed via laparoscopy with small incisions of just a few millimeters on the abdominal wall [3]. The ongoing evolution of hysterectomy techniques and the need to base surgical choices on high-quality comparative evidence have been emphasized in recent studies, calling for a broader perspective on outcomes and innovation in the field [4].

Laparoscopy is increasingly replacing open surgery as the preferred treatment option for most patients across various medical disciplines. Laparoscopic surgery offers numerous advantages over laparotomy, such as decreased postoperative intravenous analgesia requirements, shorter hospital stays, faster recovery times, and a quicker return to work and daily activities [5]. Laparoscopic hysterectomy is the most commonly performed endoscopic surgical technique worldwide [6]. Minimally invasive surgery aims to further reduce surgical trauma and the invasiveness of procedures. This can be achieved by reducing the number or size of laparoscopic access points and instruments [7], while maintaining the same high standards of surgical quality [8]. This has led to the development of minilaparoscopic surgery, which involves procedures in which the access points are 3 mm or smaller. This progress is largely due to technological advancements that have provided surgeons with smaller instruments, more powerful optics, and improved light sources [9]. Minilaparoscopy is based on the principle of minimal trauma to the abdominal wall via the use of smaller surgical instruments. This approach helps to minimize incision-related morbidity, reduce patient pain, and attenuate the stress response associated with the surgical procedure [7]. Therefore, minilaparoscopy, thanks to smaller surgical incisions, appears to result in better cosmetic outcomes [10,11] and fewer wound-related complications, such as port-site hernias.

The use of minilaparoscopy for gynecological conditions began at the end of the 20th century. Initially, it was employed mainly for diagnostic purposes or minimally invasive procedures [12]. Over the past twenty years, numerous studies have highlighted the potential of minilaparoscopic surgery for progressively complex procedures [13,14,15,16,17,18]. Today, even highly technical surgeries, such as myomectomy [19] or radical hysterectomy for cervical cancer [11], can be safely performed using 3 mm mini-instruments.

Despite the growing body of literature highlighting the potential advantages of minilaparoscopy, there remains a lack of robust comparative data specifically evaluating MLH versus conventional LH, particularly in terms of postoperative pain, operative outcomes, and complication rates. Moreover, existing studies are often limited by heterogeneity in patient populations, surgical techniques, or multicenter variability, which can affect the consistency and reproducibility of the findings.

This retrospective cohort study aims to address this gap by directly comparing operative and postoperative outcomes between MLH and LH, using data from a single Department of Gynecology. By controlling for the surgical setting and standardizing the technique, this study offers an important contribution to the current literature, providing a more homogeneous and reliable comparison between the two approaches. The findings may help refine surgical decision-making and guide future recommendations for the use of minilaparoscopy in gynecologic surgery.

## 2. Materials and Methods

In this study, patients were recruited between 1 January 2017 and 1 January 2024. The study was conducted in the Department of Gynecology at ‘Villa Sofia-Cervello’ Hospital.

Patients included in this retrospective cohort study were divided into two groups based on the surgical technique employed. The first group underwent a conventional LH, performed using standard 5 mm trocars and instruments. The second group underwent MLH, in which all operative steps were executed using 3 mm access ports and miniaturized instruments.

This retrospective cohort study aimed to compare the operative outcomes and postoperative pain of LH versus MLH using data from a single Department of Gynecology. Specifically, this study aimed to evaluate and contrast surgical duration, complication rates, pain management, and the length of hospital stay between the two approaches, with a focus on their feasibility and effectiveness in treating gynecological conditions.

### 2.1. Inclusion and Exclusion Criteria

Indications for hysterectomy included uterine myoma, endometriosis, endometrial hyperplasia, adenomyosis, high-grade cervical dysplasia, early-stage endometrial cancer, and microinvasive cervical cancer requiring type A radical hysterectomy, according to the Querleu-Morrow classification. For young patients of reproductive age who desire future childbearing and who have early-stage G1 endometrial cancer without myometrial or adnexal invasion, a fertility-sparing approach could be considered [19,20,21,22,23,24,25]. While acknowledging this approach, in our study we chose to include only patients who underwent hysterectomy.

The exclusion criteria included the need for comprehensive surgical staging of malignant disease, a previous midline laparotomy, suspected or known severe endometriosis, and myomas larger than 10 cm.

### 2.2. Operative Technique

**Figure 1:**
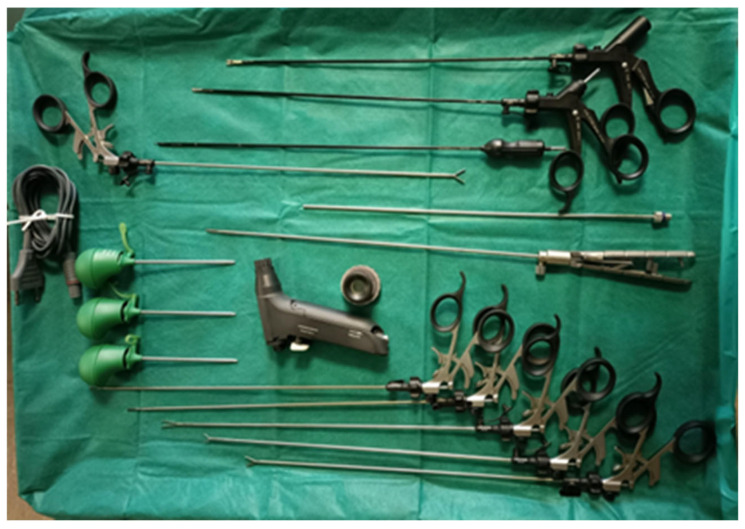
Minilaparoscopic instruments.

A standardized anesthetic protocol was used for all patients, which included induction with propofol (2–3 mg/kg) and fentanyl (1.5 mg/kg), neuromuscular blockade with midarine (1 mg/kg) and nimbex (0.15 mg/kg), and maintenance with fentanyl. Postoperatively, patients were managed with intravenous paracetamol (1 g every 8 h, starting approximately 30 min before the end of surgery) and intravenous ketorolac (every 12 h). Antibiotic prophylaxis consisted of a single dose of intravenous cefazolin (2 g), administered 30 min before the induction of anesthesia. All subjects undergoing surgery were treated by the same surgical team, which had over 10 years of laparoscopic experience. The same surgical technique was used for both types of procedures. The surgical instruments consisted of 5 mm and 3 mm graspers, scissors, monopolar electrocautery, and a suction-washing system, as shown in Figure 1. The bipolar forceps for dissection were available in both 3 mm and 5 mm versions (Ab Medica s.p.a., Milan, Italy) or the 5 mm instruments.

For both groups, an IMAGE1 S™ Rubina^®^–4K, 3D, NIR/ICG laparoscopic column was used. An intrauterine manipulator (Clermont-Fd uterine mobilizer, Karl Storz GmbH & Co., KG, Rome, Italy) was inserted, except in patients with early-stage endometrial or microinvasive cervical cancer. Patients with early-stage endometrial cancer underwent sentinel lymph node mapping, receiving cervical injections of 25 mg of indocyanine green powder mixed with 20 mL of sterile water (1.25 mg/mL): 1 mL was injected superficially (1–3 mm) and 1 mL deep (1–2 cm) at the 3 and 9 o’clock positions, for a total of 4 mL per side, using a 22-gauge spinal needle.

The lymphatic channels were stained green, allowing the surgeon to trace these channels to the sentinel lymph nodes, which were then dissected and carefully removed. This technique requires a thorough understanding of retroperitoneal anatomy to dissect the Latzko and Okabayashi spaces.

After pneumoperitoneum was established, one optical trocar (5 mm for mini-LPS and 10 mm for LPS) was introduced at the umbilical site. Under direct visualization, three ancillary trocars were inserted: two in the iliac fossas, outside the outer edge of the rectus abdominis muscle, level with or above the anterosuperior iliac spine, and one in the midline (at least 8 to 10 cm from the umbilical trocar). During the procedures, pneumoperitoneum was maintained using an insufflation system, delivering carbon dioxide through the umbilical port. The hysterectomy began with the coagulation and division of the round ligaments, ensuring sufficient distance from the uterus. The anterior and posterior folds of the broad ligament were separated. A peritoneal window was then created in the broad ligament, and the position of the ureter was checked to ensure that it was away from the dissection area. The infundibulopelvic ligaments (in cases of concomitant salpingo-oophorectomy) or the utero-ovarian ligament and mesosalpinx (in cases of ovarian preservation) were coagulated and dissected. The anterior fold of the broad ligament was opened.

The vesico-uterine junction, identified as a white line approximately 2–3 cm between the uterus and bladder, was then incised, allowing for the caudal reflection of the bladder. This step opened the vesico-uterine space. The cardinal and uterosacral ligaments were coagulated and transected. After identification of the ureter, the uterine arteries were skeletonized, coagulated, and dissected.

A circular colpotomy was then performed using a monopolar hook, and the uterus was extracted through the vagina. The vaginal vault was carefully sutured laparoscopically using two or three stitches of polyglecaprone 25 (Monocryl^®^ 1), ensuring the adequate inclusion of both the vaginal mucosa and fascia. The pelvis was irrigated, and the ureters, vaginal vault, and bladder were thoroughly checked. The trocars were removed under direct visualization, the pneumoperitoneum was completely evacuated, and the skin incisions were closed.

### 2.3. Data Collection

Data on the surgical procedures, as well as intraoperative and postoperative outcomes for both study groups, were retrospectively documented in our database. The duration of the surgery was measured from the insertion of the Veres needle. The procedure was considered converted from the mini-LPS approach to the LPS technique if any of the 3 mm ports were replaced with a 5 mm port. Postoperative pain was assessed at 6, 12, and 24 h after surgery using a 100 mm visual analog scale (VAS) [26]. Patients were asked to rate their abdominal pain at rest. Intraoperative complications were evaluated using the Satava-Kazaryan classification [27,28]. Increases in white blood cell count and drops in hemoglobin from before the operation to postoperative day 1 were calculated. Leukocytosis was considered clinically relevant if white blood cell count exceeded 13,000/µL beyond 48 h post-surgery or in association with systemic signs of infection (fever, tachycardia, or neutrophilia). A decrease in hemoglobin was considered pathological when it was >2.0 g/dL from baseline or when postoperative hemoglobin fell below 10 g/dL, particularly if associated with symptoms or hemodynamic instability. Postoperative complications were defined as negative events occurring within 30 days following surgery, directly resulting from the procedure. Febrile morbidity was defined as having two temperatures ≥ 38 °C, recorded 6 h apart, within 48 h after surgery, necessitating antibiotic therapy. The length of hospital stay was measured from the first postoperative day.

### 2.4. Data Analysis

Preliminary analyses (using means, standard deviations, and percentages) were performed in order to compare the two treatment groups according to women’s baseline characteristics. The Student *t*-test and the chi2 test for independent samples were used for continuous and categorical variables, respectively, to assess the statistical significance of differences at baseline.

In addition, a multivariable regression technique was implemented to assess the effects of treatments on outcomes (surgery duration, blood loss, hospitalization, and VAS score at 6, 12 and 24 h). In particular, linear regression models were used to establish the relationship between the type of intervention (considered independent variable) and each of the outcomes (separately considered separately dependent variables). Only variables that were unbalanced between the two treatment groups at baseline (i.e., BMI, previous CT, and previous abdominal surgery) were included as covariates in the models (one per outcome), to control for confounding (Supplementary Materials).

All statistical analyses were conducted using STATA software version 11.0 (StataCorp LLC, College Station, TX, USA).

The primary aim of this study was to compare MLH and LH in terms of surgical feasibility. The primary endpoint was surgery duration, which would highlight any differences between the two minimally invasive techniques. The secondary objective was to compare the surgical outcomes of mini-LPS and LPS using the following secondary endpoints: postoperative pain, conversion rate, blood loss (both in terms of intraoperative blood loss and hemoglobin drop post-surgery), intraoperative complications (evaluated using the Satava-Kazaryan classification) [27,28], postoperative complications (assessed with the Clavien-Dindo classification), and the length of hospital stay.

## 3. Results

During the study period, a total of 412 patients underwent hysterectomy at our department. Of these, 308 were enrolled in this study, divided into two groups: the LH group (*n* = 153) and the MLH group (*n* = 155). The demographic characteristics of the two groups are specified in Table 1.

Conversion from minilaparoscopy to the conventional laparoscopic technique occurred in 12 cases, due to dense pelvic adhesions in some patients and a large or fixed uterus in others. Replacing the 3 mm instrument with a 5 mm instrument allowed the successful completion of the procedure.

The average surgery duration was 98.8 min (SD = 1.1) for the minilaparoscopic group and 105.5 min (SD = 1.1) for the conventional laparoscopic group, as shown in Table 2. Although smaller instruments can be associated with technical challenges and longer operative times, our team was able to achieve these results thanks to over a decade of laparoscopic experience. This level of expertise allowed us to minimize time differences between the two approaches.

Intraoperative blood loss was estimated by measuring the amount of blood drained during the procedure using a graduated container and averaged 100.3 mL (SD = 4.0) in the minilaparoscopic group and 195.1 mL (SD = 5.1) in the conventional laparoscopic group. The average length of hospital stay was 3.2 days (DS = 0.1) for women undergoing minilaparoscopic procedures, compared to 4.0 days (DS = 0.1) for women undergoing conventional laparoscopy.

The pain scores for both study groups are displayed in Table 2. Pain was also evaluated by comparing the patients’ requests for analgesics between the two groups postoperatively.

## 4. Discussion

Over the past two decades, laparoscopy has gained increasing recognition for its clinical value and applicability. Over the past few years, many improvements have occurred in endoscopic surgery to further minimize surgical trauma and the invasiveness of their procedures, mainly in three major directions: one is reducing the number of access ports (such as in single-port surgery) [9,29,30], another is utilizing natural orifice translumenal endoscopic surgery [31], and the third is decreasing the diameter of trocars from 5–10 mm or more to 3 or 2 mm (as seen in mini- or microlaparoscopy) [32,33,34].

A consensus has not yet been reached on the definition of the term “minilaparoscopy” in the literature. According to many authors, it would be more appropriate to use this term exclusively for laparoscopic procedures performed entirely with 3 mm trocars, with the only possible exception being the umbilical port [7,34].

Minilaparoscopic surgery was introduced several years ago, but initially, it was reserved for diagnostic procedures only [33]. The interventional use of 3 mm instruments was primarily considered as a complementary addition to standard 5–10-mm trocars.

In the early stages, minilaparoscopic instruments posed challenges due to poor visibility, loose grasping, easy bending, inadequate irrigation or suction, and reduced durability [35]. However, with advancements in surgical endoscopic techniques, as well as improvements in equipment and materials, a significant number of procedures have become feasible using minilaparoscopic instruments alone. This progress has been made possible by technological advancements that have led to a broad range of minilaparoscopic instruments, such as bipolar graspers, dissectors, needle holders, and suction-irrigation devices, coupled with better training for surgeons. Consequently, the applicability of minilaparoscopic surgery has expanded across many gynecological conditions, which is especially important for patients seeking improved cosmesis [36].

The use of smaller-caliber instruments has not diminished the ability of gynecologic surgeons to perform technically demanding procedures. Compared with other minimally invasive techniques, minilaparoscopy has the advantage of maintaining the same ergonomics and instrument types as standard laparoscopy. As a result, for experienced laparoscopists, mastering this new technique is not problematic.

There are several potential benefits of minilaparoscopy compared to standard laparoscopy, including reduced peri- and postoperative pain [26,37] and improved cosmetic satisfaction, due to the use of 3 mm trocars. Additionally, reducing the size of the wound on the abdominal wall may be associated with a decreased risk of incisional hernias [38], a rare but potentially serious complication. It has been observed that as the size of laparoscopic access ports increases, so does the risk of incisional hernias [35].

In gynecological surgery, the first applications of this technique focused on adnexal procedures, followed by total hysterectomy [11], and later extended to the treatment of endometrial, cervical [36], and ovarian cancers [14,18], as well as standard hysterectomies [7,39,40]. Surgeons then progressed to intraperitoneal lymphadenectomy [40], and, at present, total minilaparoscopic radical hysterectomy [7,39,40,41] is being performed.

The introduction of 3 mm trocars for laparoscopic hysterectomy procedures has proven to be as effective as using standard-caliber trocars [7,42,43,44]. The development of an efficient 3 mm bipolar instrument has been crucial in these advances, as it has allowed for the adequate control of hemostasis in medium-sized vessels, such as uterine arteries, thus preventing potential major complications.

As numerous studies have shown, the miniaturization of instruments has maximized the well-known benefits of standard minimally invasive techniques, such as smaller surgical scars, reduced blood loss, less postoperative pain, shorter hospital stays, quicker recovery times, and a lower risk of infection and herniation [42,45,46].

Over the years, several randomized studies have compared traditional laparoscopy with the use of smaller-caliber instruments and access ports. The results have not been consistent. In fact, some studies have shown that reducing the number or size of laparoscopic access ports significantly reduces postoperative pain [45,46]. Many other studies, in addition to ours, have demonstrated the superiority of the minimally invasive technique compared to the conventional one. The results are similar, with a reduction in bleeding and complications [2]. However, other studies have failed to demonstrate a significant reduction in postoperative pain or the length of hospital stay [47,48].

Another key aspect to consider, particularly in light of the distinct characteristics of each patient and the complexities involved in outlining a therapeutic surgical approach, is the role of evidence-based guidelines and best practices. Viable treatment options should be thoroughly discussed with patients, ensuring that their individual needs are met with clear and comprehensive explanations. This consultation should also include an evaluation of potential complications, as many cases of negligence, malpractice claims [49,50,51], and lawsuits involving laparoscopic hysterectomy procedures stem from damage to the ureter. Recent research has highlighted the importance of identifying predictors of both overall and severe postoperative complications, particularly in oncologic gynecological surgery, where patient fragility plays a significant role in outcomes [52]. In particular, a study by Restaino et al. emphasized the clinical relevance of assessing the fragility index preoperatively, suggesting that it may serve as a valuable tool to anticipate postoperative risks and improve individualized perioperative management strategies. It is therefore crucial to raise awareness among patients about these complications and their potential consequences, as well as available alternatives.

For example, a 2011 lawsuit involved a patient with menorrhagia who, after consulting with her gynecologist, underwent a laparoscopic hysterectomy [50]. The court found that no alternative options were discussed with the patient, despite the fact that three potential alternatives could have been considered: symptom management with medication, the insertion of a Mirena Coil [53], or uterine embolization. Additionally, the risks of the procedure were not adequately explained, preventing the patient from making an informed decision regarding the therapeutic pathway to pursue. This failure undermined the informed consent process. More recent court cases have resulted in significant compensatory damages, reflecting the severe and life-altering impact of complications, including those that arose after emergency surgery to treat damage to the ureters and bladder. One case involved complications such as a fistula, kidney infections, recurrent urinary tract infections (UTIs), and loss of bladder sensation. Ultimately, a USD 1.5 million out-of-court settlement was reached [54].

These litigation outcomes highlight how malpractice in hysterectomy can lead to damages that, while not life-threatening, can significantly affect quality of life—not only for patients but also for their families, with far-reaching medical, legal, and ethical implications [50]. This underscores the importance of ensuring that informed consent is properly administered and signed, both to guide the patient appropriately and to avoid potential medical disputes that benefit neither the patient, the physician, nor society at large [51,52,53,54]. Such cases can involve not only physicians and healthcare facilities but also device manufacturers, particularly in cases of design defects or insufficient warnings regarding the risks associated with these procedures.

Furthermore, minimally invasive 360-degree laparoscopic techniques are not the final stage of innovation. Unprecedented challenges are expected to arise from scientific and technological advancements, which are poised to profoundly alter the implementation of healthcare and the conception of patient care [55,56,57]. These include, for instance, AI-powered diagnostics in gynecology and beyond [58], as well as robotic surgery. These innovations have led to an urgent need to redefine the legal, regulatory, and ethical frameworks upon which healthcare and research have long been based. Research has revealed the still poorly defined ethical, legal, and social implications of such innovations, three of which are particularly noteworthy: first, there is a lack or inadequacy of standardized training and practice in robotic surgery and AI/big data management, which may pose significant risks to both patient safety and surgical expertise; second, as mentioned earlier, the informed consent process, which is essential for medicolegal validity, will require substantial modification to ensure that patients are fully aware of these innovative technologies and their capabilities, strengths, and limitations; third, the concept of legal liability itself will become more complex due to the functional characteristics of robotic systems, which require the involvement of multiple new operators and stakeholders, including the manufacturers of increasingly complex systems. As these systems gain greater autonomy, and as developments in artificial intelligence and machine learning continue [59,60], new ethical, legal, and liability dilemmas are likely to emerge.

Finally, gynecological oncological diseases, like many other stressful conditions, pose a challenge not only physically but also psychologically, as they deeply impact self-perception, including that regarding femininity, and quality of life. They often generate feelings of anxiety, fear of the future, and social isolation, necessitating multidisciplinary support to ensure the emotional well-being of patients [61,62,63,64,65].

Additionally, these conditions may exacerbate pre-existing vulnerabilities or mental health issues, further underlining the importance of comprehensive care that addresses both the physical and emotional aspects of the disease.

The psychological aspect of these gynecological procedures cannot be overlooked. The choice of a minimally invasive surgical technique can indeed impact a woman’s well-being, influencing her experience and adding an emotional dimension that is not entirely negative. Laparoscopic surgery, in fact, uses small incisions on the skin compared to conventional methods, which allows for quicker recovery. Additionally, the thought of not having large scars helps women alleviate the stress of the surgical procedure. Therefore, it can be concluded that laparoscopic surgery not only provides positive physical results in terms of pain but also, from a psychological standpoint, helps patients relate better to their condition [59]. This also benefits healthcare providers, who can interact with patients without them being entirely overwhelmed by psychological concerns.

Although our study primarily focuses on two techniques, it is still important to mention robotic and vaginal hysterectomy, as these are becoming increasingly relevant in the field of gynecological surgery, representing a significant advancement in surgical approaches, with benefits for patients in terms of precision, recovery times, and a reduction in complications [65,66,67]. We do not discuss these methods in detail in order to stay focused on the scope of our study, but we hope to be able to examine and analyze them more thoroughly in future studies, which will allow us to explore them with greater attention.

## 5. Conclusions

In conclusion, this study has demonstrated the feasibility and excellent functionality of mini-LPS-H in the treatment of uterine fibroids, endometriosis, endometrial hyperplasia, adenomyosis, high-grade cervical dysplasia, early-stage endometrial cancer, and microinvasive cervical cancer. These results are supported by key outcomes including surgical duration, the adequacy of surgical specimens for histopathological analysis, conversion rate, blood loss, postoperative complications, and the length of hospital stay. The study also highlighted the superiority of mini-LPS in terms of pain, which was higher in the first few hours following the procedure and slightly lower after 24 h. The findings of this study provide a strong foundation for future investigation and may contribute to shaping the next generation of gynecologic surgical standards. Future research should focus on validating these results through multicenter, prospective, randomized, controlled trials with larger and more diverse patient populations. Long-term follow-up is also warranted to assess outcomes related to recurrence, quality of life, patient satisfaction, and postoperative functional recovery. Additional studies should explore cost-effectiveness, standardized pain, and cosmetic assessment tools. Evidence-based clinical practices must, of course, be grounded in standards and guidelines that account for the pace and level of innovation in the medical field, which could lead to significant advancements in hysteroscopy, other gynecological procedures, and healthcare.

## Figures and Tables

**Table 1:** Baseline characteristics of each group.

	LH Group (*n* = 153)	MLH Group (*n* = 155)	*p*-Value
Age (mean ± SD)	50.0 (±0.6)	49.5 (±0.6)	n.s. *
BMI(mean ± SD)	27.1 (±0.5)	28.5 (±0.4)	<0.05
Menopause (%)	79.7	46.5	<0.0001
Parity (mean ± SD)	2.1 (±0.1)	2.0 (±0.1)	n.s. *
Previous caesarian section (%)	55.6	22.6	<0.0001
Previous abdominal surgery (%)	20.9	45.8	<0.0001

* n.s.: not significant.

**Table 2:** Comparative analysis of minilaparoscopic surgery vs. conventional laparoscopy outcomes.

Surgical Outcomes	LH Group	MLH Group	Coeff. (MLH vs. LH) *	Std. Error	*t*	*p*-Value	Adj R^2^	CI95% Lower Lim.	CI95% Upper Lim.
Surgery duration (mean ± SD)	105.5 min (±1.2)	98.8 min (±1.1)	−6.7	1.835	−3.65	<0.0001	0.0395	−10.3	−3.1
Blood loss (mean ± SD)	195.1 (±5.1)	100.3 (±4.0)	−94.2	7.095	−13.28	<0.0001	0.4049	−108.2	−80.2
Length of hospital stay (mean ± SD)	4.0 (±0.1)	3.2 (±0.1)	−0.9	0.089	−9.72	<0.0001	0.2518	−1.0	−0.7
VAS score at 6 h (mean ± SD)	7.0 (±0.06)	7.9 (±0.06)	0.9	0.086	10.75	<0.0001	0.2915	0.8	1.1
VAS score at 12 h (mean ± SD)	6.0 (±0.04)	5.5 (±0.06)	−0.5	0.087	−5.67	<0.0001	0.1198	−0.7	−0.3
VAS score at 24 h (mean ± SD)	4.2 (±0.04)	4.1 (±0.03)	−0.09	0.054	−1.55	>0.10	0.0054	−0.19	+0.02

(*) Regression coefficients adjusted for patients’ clinical conditions at baseline (BMI value, previous CT scan, previous abdominal surgery), using multiple regression model.

## Data Availability

All data are available upon request from the corresponding author.

## Supplementary Materials

The following supporting information can be downloaded at: https://www.mdpi.com/article/10.3390/medicina61071216/s1, File S1: Multivariable regression technique.
