# Supplementary material for: Minilaparoscopic Versus Conventional Laparoscopic Hysterectomy: Insights from a Single-Center Retrospective Cohort Study with Legal Considerations
Source: Medicina (Kaunas). 2025 Jul 3;61(7):1216. doi: 10.3390/medicina61071216 (PMC12298684; doi:10.3390/medicina61071216)
Supplement: Supplementary file 1 [file medicina-61-01216-s001.zip › medicina-3654332-supplementary.pdf]

File S1

Linear regression models

Surgery duration

xi: regress sur_duration i.MDL-LH i.CT_scan i.pre_surgery BMI

Source | SS df MS Number of obs = 308

-------------+------------------------------ F(4, 303) = 4.16

Model | 3617.43471 4 904.358677 Prob > F = 0.0027

Residual | 65,936.8348 303 217.613316 R-squared = 0.0520

-------------+------------------------------ Adj R-squared = 0.0395

Total | 69,554.2695 307 226.561138 Root MSE = 14.752

Sur_duration | Coef. Std. Err. t P > |t| [95% Conf. Interval]

-------------+--

MDL (vs. *LH) | −6.690618 1.83469 −3.65 0.000 −10.30096 −3.080271*

CT_scan | 0.6337811 1.849866 0.34 0.732 −3.006429 4.273991

Pre_surgery | 0.0668845 1.867842 0.04 0.971 −3.608699 3.742468

BMI | 0.11075 0.1519881 0.73 0.467 −0.1883359 0.4098358

_cons | 102.1135 4.443426 22.98 0.000 93.36967 110.8574

Blood loss

xi: regress blood_loss i.MDL-LH i.CT_scan i.pre_surgery BMI

Source | SS df MS Number of obs = 308

-------------+------------------------------ F(4, 303) = 53.23

Model | 692,986.496 4 173,246.624 Prob > F = 0.0000

Residual | 986,183.309 303 3254.73039 R-squared = 0.4127

-------------+------------------------------ Adj R-squared = 0.4049

Total | 1,679,169.81 307 5469.60849 Root MSE = 57.05

----

blood_loss | Coef. Std. Err. t P > |t| [95% Conf. Interval]

-------------+--

MDL (vs. LH) | −94.20362 7.095408 −13.28 0.000 −108.1661 −80.2411

CT_scan | 2.517725 7.154099 0.35 0.725 −11.56028 16.59573

Pre_surgery | 0.436248 7.223618 0.06 0.952 −13.77856 14.65106

BMI | 0.058776 0.5877929 0.10 0.920 −1.097897 1.215449

_cons | 192.0173 17.18433 11.17 0.000 158.2015 225.833

Length of hospital stay

xi: regress length_stay i.MDL-LH i.CT_scan i.pre_surgery BMI

Source | SS df MS Number of obs = 308

-------------+------------------------------ F(4, 303) = 26.83

Model | 55.2655419 4 13.8163855 Prob > F = 0.0000

Residual | 156.016926 303 0.514907345 R-squared = 0.2616

-------------+------------------------------ Adj R-squared = 0.2518

Total | 211.282468 307 0.688216507 Root MSE = 0.71757

----

length_stay | Coef. Std. Err. t P > |t| [95% Conf. Interval]

-------------+--

*MDL (*vs. *LH) | −0.8672069 0.0892451 −9.72 0.000 −1.042826 −0.6915882*

CT_scan | −0.0321944 0.0899833 −0.36 0.721 −0.2092657 0.1448769

Pre_surgery | 0.0241217 0.0908577 0.27 0.791 −0.1546703 0.2029137

BMI | 0.0037396 0.0073932 0.51 0.613 −0.0108089 0.0182881

_cons | 3.911627 0.2161423 18.10 0.000 3.486298 4.336957

VAS score at 6 h

xi: regress vas_6h i.MDL-LH i.CT_scan i.pre_surgery BMI

Source | SS df MS Number of obs = 308

-------------+------------------------------ F(4, 303) = 32.57

Model | 62.6235771 4 15.6558943 Prob > F = 0.0000

Residual | 145.645903 303 0.480679549 R-squared = 0.3007

-------------+------------------------------ Adj R-squared = 0.2915

Total | 208.269481 307 0.678402217 Root MSE = 0.69331

----

vas_6h | Coef. Std. Err. t P > |t| [95% Conf. Interval]

-------------+--

*MDL (*vs. *LH) | 0.9271614 0.0862279 10.75 0.000 0.7574801 1.096843*

CT_scan | 0.1043313 .0869411 1.20 0.231 −0.0667536 0.2754161

Pre_surgery | 0.0200936 0.087786 0.23 0.819 −0.1526537 0.192841

bmi | −0.0006794 0.0071432 −0.10 0.924 −0.014736 0.0133772

_cons | 6.956225 0.2088349 33.31 0.000 6.545274 7.367175

VAS score at 12 h

xi: regress vas_12h i.MDL-LH i.CT_scan i.pre_surgery BMI

Source | SS df MS Number of obs = 308

-------------+------------------------------ F(4, 303) = 11.45

Model | 22.1724643 4 5.54311607 Prob > F = 0.0000

Residual | 146.684679 303 0.48410785 R-squared = 0.1313

-------------+------------------------------ Adj R-squared = 0.1198

Total | 168.857143 307 0.550023267 Root MSE = 0.69578

----

vas_12h | Coef. Std. Err. t P > |t| [95% Conf. Interval]

-------------+--

*MDL (*vs. *LH) | −0.4904963 0.0865348 −5.67 0.000 −0.6607816 −0.320211*

CT_scan | 0.0856987 0.0872506 0.98 0.327 −0.0859951 0.2573926

Pre_surgery | −0.0242476 0.0880985 −0.28 0.783 −0.1976099 0.1491146

BMI | −0.0027491 0.0071687 −0.38 0.702 −0.0168558 0.0113576

_cons | 6.012259 0.2095783 28.69 0.000 5.599845 6.424672

VAS score at 24 h

xi: regress vas_24h i.MDL-LH i.CT_scan i.pre_surgery BMI

Source | SS df MS Number of obs = 308

-------------+------------------------------ F(4, 303) = 1.42

Model | 1.07288687 4 0.268221719 Prob > F = 0.2282

Residual | 57.3524378 303 0.189281973 R-squared = 0.0184

-------------+------------------------------ Adj R-squared = 0.0054

Total | 58.4253247 307 0.190310504 Root MSE = 0.43507

----

vas_24h | Coef. Std. Err. t P > |t| [95% Conf. Interval]

-------------+--

MDL (vs. *LH)* | *−0.0837883 0 .0541096 −1.55 0.123 −0.1902665 0.0226899*

CT_scan | *0.0465571 0.0545572 0.85 0.394 −0.0608018 0.1539161*

Pre_surgery | *0.0229568 0.0550874 0.42 0.677 −0.0854454 0.131359*

bmi | *−0.0040517 0.0044825 −0.90 0.367 −0.0128725 0.0047691*

_cons | *4.275072 0.1310478 32.62 0.000 4.017193 4.532951*
